# Microscopic Evidence of Haze Formation During the COVID-19 Lockdown in Beijing: Insights from Physicochemical Properties

**DOI:** 10.3390/toxics13121051

**Published:** 2025-12-04

**Authors:** Wenjun Li, Longyi Shao, Timothy P. Jones, Hong Li, Daizhou Zhang, Weijun Li, Jian Gao, M. Santosh, Shushen Yang, Kelly BéruBé

**Affiliations:** 1 State Key Laboratory of Environmental Criteria and Risk Assessment, Chinese Research Academy of Environmental Sciences, Beijing 100012, China; li.wenjun@craes.org.cn (W.L.); lihong@craes.org.cn (H.L.); gaojian@craes.org.cn (J.G.); 2 College of Geoscience and Surveying Engineering, China University of Mining and Technology (Beijing), Beijing 100083, China; 3 School of Earth and Environmental Sciences, Cardiff University, Cardiff CF10 3YE, Wales, UK; jonestp@cardiff.ac.uk; 4 Faculty of Environmental and Symbiotic Sciences, Prefectural University of Kumamoto, Kumamoto 862-8502, Japan; dzzhang@pu-kumamoto.ac.jp; 5 Department of Atmospheric Sciences, School of Earth Sciences, Zhejiang University, Hangzhou 310027, China; liweijun@zju.edu.cn; 6 School of Earth Sciences and Resources, China University of Geoscience (Beijing), Beijing 100083, China; santosh@cugb.edu.cn; 7 Department of Earth Science, University of Adelaide, Adelaide, SA 5005, Australia; 8 Department of Environment Engineering, Zhongyuan University of Technology, Zhengzhou 450007, China; yangss@zzti.edu.cn; 9 School of Biosciences, Cardiff University, Cardiff CF10 3AX, Wales, UK; berube@cardiff.ac.uk

**Keywords:** COVID-19, individual aerosol particles, physicochemical properties, secondary aerosol formation, TEM-EDX

## Abstract

The COVID-19 pandemic emerging in early 2020 triggered global responses. In China, stringent lockdown measures were implemented to suppress the rapid spread of infection, resulting in substantial reductions in anthropogenic emissions. However, several atmospheric haze episodes still occurred. Previous studies have investigated the cause of these haze events predominantly based on the average concentration obtained from bulk analysis, while the micro-scale structure and composition of the haze particles remain poorly understood. In this study, we analyzed the morphology and elemental composition of individual airborne particles collected from an urban area of Beijing in early 2020 using high-resolution transmission electron microscopy equipped with Energy Dispersive X-ray Spectroscopy. The results show that sulfur-dominant, ultrafine, and mixed particles were the most abundant types during the pollution process. Reduced human activities corresponded with a lower percentage of anthropogenic-derived soot, organic particles, and metal-containing particles. Atmospheric aging analysis demonstrated that secondary aerosols were the most significant component during the haze events. The proportion of core–shell particles increased with the intensification of the pollution, while the core/shell ratio of the particles decreased, suggesting a substantial contribution of secondary aerosols to the haze formation. Despite reductions in anthropogenic emissions, larger proportions of secondary aerosol formation enhanced aerosol aging and thereby caused episodic haze pollution during the lockdown period.

## 1. Introduction

The emergence of the Coronavirus Disease 2019 (COVID-19) in early 2020 resulted in response measures worldwide and was declared to be a global public health emergency of international concern [1]. Due to its high human-to-human transmissibility, the disease rapidly spread worldwide and influenced human activities with frequent epidemic breakouts. As one of the affected countries, China promptly implemented numerous stringent lockdown measures to mitigate the transmission of the COVID-19 virus. Public transportation, educational institutes, business centers, parks, and other social sectors were partially or fully suspended, resulting in a dramatic decrease in social mixing, traffic, economy, and daily anthropogenic activities. The transportation sector was heavily impacted, experiencing a sharp decrease in the number of on-road vehicles [2,3], because people were either unwilling or restricted from traveling, leading to a significant reduction in vehicle emission sources. Industrial production and manufacturing sectors were also severely affected by the pandemic, as evidenced by the shutdown of high-polluting facilities like coal-fired power plants, which reduced coal consumption.

As a result of the response measures, anthropogenic emissions witnessed a significant decrease in most countries affected by the pandemic [4]. China, as one of the hotspots of the pandemic, particularly Wuhan, Hubei, experienced a notable air quality improvement during the COVID-19 lockdown, with remarkable reductions in PM_2.5_, PM_10_, SO_2_, CO, and NO_2_ concentrations [5]. Satellite data revealed an average 40% decrease in NO_2_ across Chinese cities [6], with Wuhan specifically experiencing 20–30% reductions after the lockdown [2]. Despite these dramatic reductions in emissions from human activities, several haze episodes still occurred in the North China Plain during the Spring Festival and Lantern Festival periods [7]. These unusual haze episodes prompted a thorough investigation to reveal the relationship between source emissions and air quality, which is crucial for effective air pollution management in megacities. However, the causes of this air pollution were complicated by its temporal coincidence with the Chinese New Year holidays.

Previous ground-level observations and model studies have elucidated key factors contributing to the severe haze formation, including local primary emissions, regional transport, secondary aerosol formation, and stagnant meteorological conditions [7,8,9]. Comprehensive measurements and modeling revealed that the haze events during the initial lockdown period were primarily driven by enhanced secondary pollution due to intensified atmospheric oxidation [9]. Primary aerosol particles are predominantly derived from direct emissions, such as vehicle exhaust, industrial processes, and biomass burning. Conversely, secondary aerosols are formed through complex atmospheric chemical reactions, notably the oxidation of volatile organic compounds (VOCs), SO_2_, and Nox, leading to the formation of secondary organic aerosol (SOA) and secondary inorganic aerosol (SIA, e.g., sulfate and nitrate). After excluding the Spring Festival periods affected by fireworks, Ma et al. [10] found that ozone levels, sulfate, and nitrate in PM_2.5_ increased, thereby enhancing atmospheric oxidation capacity and photochemistry. The response of air pollutant concentrations to emission reductions typically exhibited non-linear patterns, influenced by both meteorological conditions and complex atmospheric physicochemical processes [11].

The above studies mostly based on bulk analysis techniques, which lacked direct evidence of haze pollution achieved through morphological and chemical analysis. Individual particles of different origins vary in physicochemical properties, such as size, morphology, and chemical composition, with surface characteristics often differing substantially from bulk properties due to atmospheric surface reactions and adsorption processes [12,13,14]. Consequently, microanalytical techniques, including Scanning/Transmission Electron Microscopy equipped with Energy Dispersive X-ray Spectroscopy (SEM-EDX/TEM-EDX), can provide crucial insights into haze formation and evolution by offering detailed information on the physicochemical properties of individual particles [15].

To investigate air pollution formation at the initial stages of the COVID-19 lockdown period, ambient particle samples were collected in a residential area of Beijing in early 2020. Using TEM-EDX, we characterized variations in the physicochemical properties of individual particles caused by reduced human activities, including morphology, size distribution, and elemental compositions. Our study particularly focused on the temporal evolution and aging process of secondary aerosol during the haze episode by analyzing variations in morphological features, core–shell structured particles, and phase separations of individual airborne particles. The study can provide direct evidence of reduced anthropogenic emissions and enhanced secondary inorganic aerosol during this episodic haze.

## 2. Experimental Methods

### 2.1. Study Area

**Figure 1:**
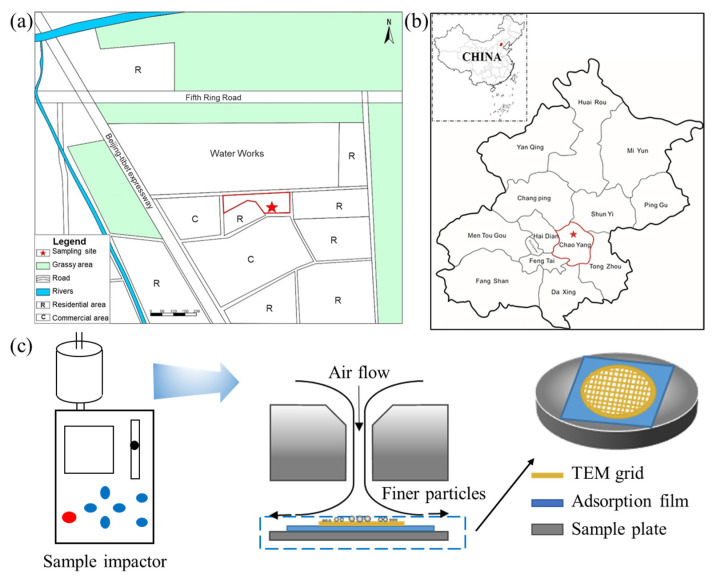
**Study area and particle sampling.** (**a**) Location of the ambient particle sampling site; (**b**) the location of the area in Beijing and the location of Beijing in China; (**c**) schematic illustration of the sample impactor and particle sampling. Note that the “Water Works” in figure (**a**) is the largest surface water plant in Beijing, which aims to purify raw surface water sourced from the South-to-North Water Diversion Project and the Miyun Reservoir to produce safe drinking water for the municipal supply network.

Particle samples were collected in a residential area (40°1′1.5″ N, 116°21′53.3″ E) in Chaoyang District, Beijing. During sampling, no confirmed COVID-19 cases were reported in the sampling area, and collection activities followed strict self-protection procedures. There were no recognizable air-polluting industrial or local pollution sources near the sampling site. The residential area is adjacent to commercial areas, located 550 m west of the Beijing-Tibet Expressway, 650 m north of the North 5th Ring Road, and 780 m east of Lincui Road (Figure 1a), representing a typical urban area of Beijing.

### 2.2. Particle Sampling

Samples were collected using a two-stage cascade impactor (DKL-2, Jinshida Inc., Qingdao, China) (Figure 1b). The impactor consists of two consecutive stages with 0.5 mm (upper stage) and 0.3 mm (lower stage) diameter jet nozzles. Airflow-entrained particles enter the sampler with coarser particles depositing on the upper stage, whereas the finer particles are diverted to the lower stage. The impactor collects particles in a size range of 0.01–10 μm [16] and was set 1.5 m above ground in an open community space. Particles were deposited onto copper TEM grids coated with carbon film (3 mm, 300-mesh copper, Tianld Co., Ltd., Beijing, China) at a flow rate of 1.0 L/min. Sampling was conducted four time daily (9:00 h, 13:00 h, 17:00 h, and 21:00 h) from February 4th to March 6th, 2020, with different sampling durations determined by real-time air quality index (AQI) and PM_2.5_ mass concentration to ensure a suitable particle density on the grids (Table 1). Meteorological parameters, including temperature and relative humidity, were recorded using a Pocket Weather Meter (Kestral 4000, Nielsen-Kellermann Inc., Boothwyn, PA, USA). The real-time PM_2.5_ and PM_10_ concentrations were monitored by a laser detector (SDL 301, Norsquare Inc., Jinan, Shandong, China). After sampling, the validity of deposited particles was verified immediately by a portable digital microscope (3R-WM601WIFI, Anyty Inc., Beijing, China). Eighty-eight samples were collected and stored in a desiccator (25 °C, 20 ± 3% relative humidity). In this study, eighteen samples collected by the upper stage (0.5 mm jet nozzles) were selected for TEM-EDX measurements. The cut-off diameter of the upper stage for approximately 100% collection efficiency is 0.5 μm for particle density of 2 g/cm^3^ [16]. Particle samples collected during a haze episode from 7 to 14 February were chosen to investigate pollution formation, alongside a “good” air quality sample (22 February) for comparison. Detailed information about samples is provided in Table 1.

### 2.3. TEM-EDX Analysis

The morphology of individual particles was characterized using a JEM-2100 LaB6 TEM (JEOL Inc., Tokyo, Japan), and elemental composition (Z ≥ 6) was measured by EDX. TEM grids were mounted onto the sample holder and introduced into the sample chamber for in situ analysis at an acceleration voltage of 200 kV. The deposited individual particles were heterogeneously distributed, with coarser particles densely deposited near the center and finer particles sparsely deposited towards the periphery. TEM images were taken from the center to the periphery to ensure representative measurements. EDX spectra were collected for 15–60 s per particle, with duration adjusted depending on particle stability under electron beam exposure. Cu signals from the TEM grids were excluded from EDX spectral analysis due to strong interference.

### 2.4. Data Analysis

TEM images were analyzed using Fiji software (based on ImageJ 1.53q) developed by National Institutes of Health (NIH, Bethesda, Maryland, USA), an open-source image processing platform [17]. All individual particles deposited on TEM grids were processed, including the nano-scale ultrafine particles (<100 nm in diameter). A total of 8091 individual particles were analyzed, with shape parameters (including area and perimeter) obtained by manually tracing the boundaries. The equivalent circular diameter (D_aeq_) was defined as the diameter of a circle with equivalent projected area to the measured particle, calculated by Equation (1).

(1) $$D_{aeq}=2\sqrt{\frac{A}{\pi }}$$

where D_aeq_ is the equivalent circular diameter (μm), and A represents the projected area (μm^2^) measured via ImageJ image processing.

To assess air quality variations at the sampling site, hourly air quality monitoring data were obtained from the nearest national-controlled station (Olympic Center). The hourly concentrations of PM_2.5_, SO_2_, NO_2_, CO, and O_3_ during the observation period were downloaded from the website (https://quotsoft.net/air/, accessed on 3 August 2020). The air quality index (AQI) was subdivided into six health risk levels, including good (AQI < 50), moderate (AQI: 51–100), unhealthy for sensitive groups (AQI: 101–150), unhealthy (AQI: 151–200), very unhealthy (AQI: 201–300), and hazardous (AQI > 300). Hourly meteorological data, including temperature, relative humidity, wind speed and direction, atmospheric pressure, and visibility, were downloaded from the website (https://airwise.hjhj-e.com/, accessed on 3 August 2020) to evaluate potential influences on haze formation.

## 3. Results

### 3.1. Air Pollution Processes

**Figure 2:**
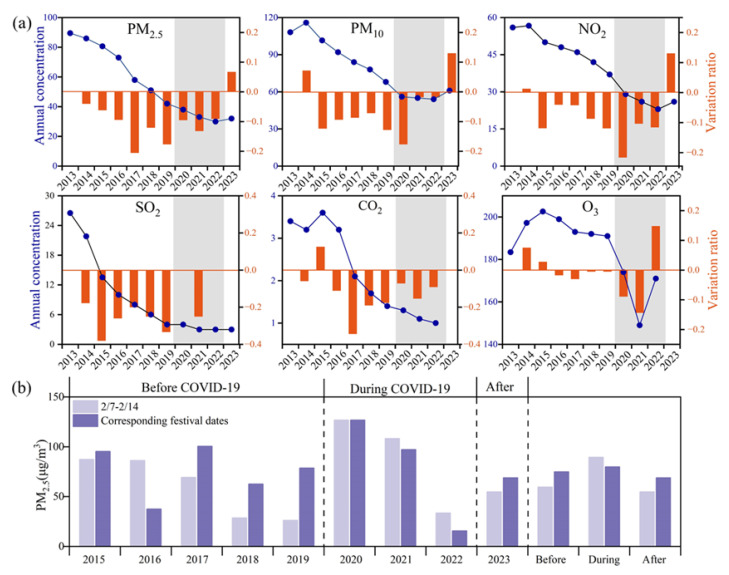
The long-term variations in air pollutants. (**a**) The variations in the annual concentrations of six conventional pollutants (blue line) and the variation ratio (red bar) during 2013–2023 in Beijing. Note that the gray area represents the three years influenced by the COVID-19 epidemic control policy; (**b**) the comparison of PM_2.5_ concentrations during the observation period (from 7 to 14 February) and the corresponding festival dates during 2015–2023.

**Figure 3:**
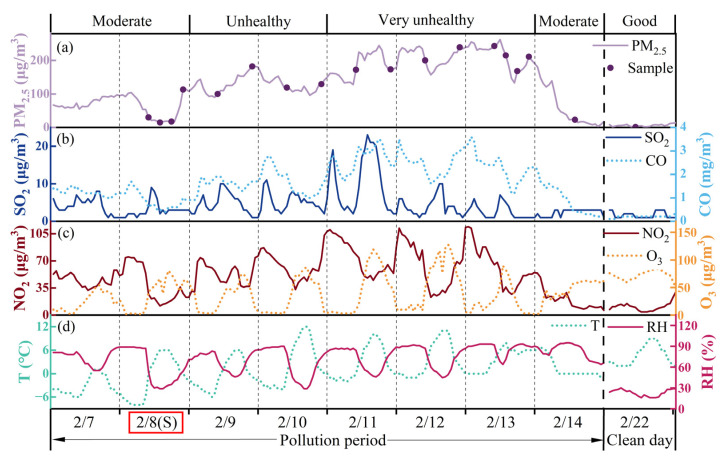
The variations in pollutant concentrations and meteorological factors at the sampling site during the research period in 2020. (**a**) the variation in PM_2.5_ concentration; (**b**) the variations in SO_2_ and CO concentrations; (**c**) the variation in NO_2_ and O_3_ concentrations; (**d**) the variations in relative humidity (RH) and temperature (T). Note that the purple dot in Figure 1a represents the collection time of each sample. The notation at the top means the air quality level. The (S) notation of 8 February marked with a red box in the x-axis represents the day of the Lantern Festival.

The impact of COVID-19 in China began in early 2020 and lasted until late 2022. During these three years, Beijing exhibited notable reductions in most air pollutants, with NO_2_ showing the most significant decrease, followed by reductions in PM_2.5_, CO, SO_2_, PM_10_, and O_3_ (Figure 2a). Despite these reductions in anthropogenic pollutants, several haze episodes still occurred during the initial stage of the COVID-19 lockdown period in the North China Plain, including a haze event lasting from 8 to 14 February 2020 (Figure 3). This haze event coincided with the Lantern Festival, which fell on 8 February 2020, the fifteenth day of the Chinese New Year holidays, and also the final day for permitted fireworks in designated areas of Beijing. According to Chinese culture and traditional customs, fireworks and firecrackers are set off during the Chinese New Year holidays for celebration in specific areas, particularly on New Year’s Eve and on the first and the fifteenth day of the festival. PM_2.5_ concentrations dramatically increased on the afternoon of the Lantern Festival, causing air quality to deteriorate from “moderate” to “unhealthy” overnight. Source apportionment research attributed the onset of this haze event primarily to fireworks and residential burning emissions (33%), with elevated levels of firework tracer species (SO_2_, Cl^−^, K^+^, and Mg^2+^) and CO detected in PM_2.5_ [7]. Air pollutants continued to accumulate from 9 to 10 February, leading to further deterioration in air quality on 11 February, when hourly PM_2.5_ concentrations peaked at 262 μg/m^3^, directly elevating the air quality to “very unhealthy”. The haze event concluded on 14 February, following a heavy snowfall at noon on 13 February, which caused a sharp decline in PM_2.5_ concentrations and restored air quality to “moderate” levels.

To further investigate the impact of fireworks and firecrackers during Spring Festival celebrations, the average PM_2.5_ concentrations from 7 to 14 February were compared to those during the corresponding festival dates between 2015 and 2023 (Figure 2b). Over the nine years, the average PM_2.5_ concentrations from 7 to 14 February were the highest recorded in 2020. The results revealed higher PM_2.5_ concentrations during festival dates (averaged 75.9 μg/m^3^) than those from 7 to 14 February (averaged 69.0 μg/m^3^) during 2015–2023, demonstrating the contribution of fireworks and firecrackers during the Lantern Festival. There were three distinct phases during the nine years, including pre-pandemic (2013–2019), pandemic (2020–2022), and post-pandemic periods (2023). Pre-pandemic years mostly showed consistently higher PM_2.5_ concentrations during festival dates than those from 7 to 14 February, due to unrestricted fireworks and firecrackers. However, the pandemic periods reversed this trend, with festival dates showing lower PM_2.5_ concentrations than those from 7 to 14 February due to restricted celebrations and reduced human activities. The post-pandemic year rebounded with higher PM_2.5_ concentrations during the festival dates, confirming restored fireworks and firecrackers. This analysis proves the substantial influence of anthropogenic activities in driving air pollution, particularly fireworks and firecrackers for festival celebrations, with the pandemic providing a unique natural experiment to isolate this impact.

### 3.2. Individual Particle Types

**Figure 4:**
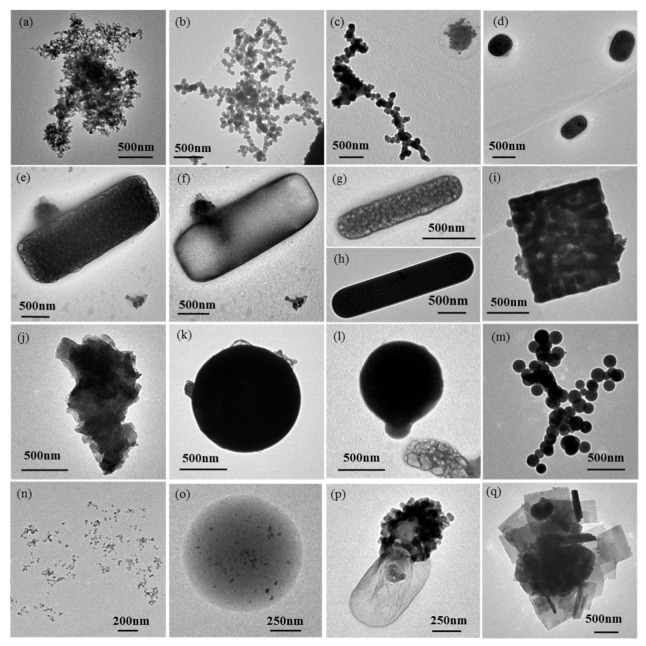
Types of individual ambient particles. (**a**) soot particle: compact soot; (**b**) soot particle: cluster soot; (**c**) soot particle: chain-like soot; (**d**) sulfur-dominant particle: elliptic sulfate particle; (**e**) sulfur-dominant particle: rod-like sulfate particle with soot particle externally mixed; (**f**) residue of particle “e” after exposure under the electron beam; (**g**) sulfur-dominant particle: rod-like sulfate with an OM coating; (**h**) sulfur-dominant particle: rod-like sulfate without an OM coating; (**i**) sulfur-dominant particle: squarely shaped sulfate; (**j**) mineral particle; (**k**) organic (OM) particle: tarball; (**l**) organic (OM) particle: tarball after electron beam damage with the vaporized sulfate in the bottom right; (**m**) metal-containing particle: Fe spheres aggregate; (**n**) ultrafine particle; (**o**) droplet-like particle; (**p**) mixed particle: external mixture of sulfate and soot; and (**q**) mixed particle: internal mixture of sodium salts and mineral particle.

According to the morphological characteristics, elemental composition, and stability under the electron beam, a total of 8091 individual particles were classified into eight types: soot (2.9%), sulfur-dominant particles (51.5%), mineral particles (3.3%), organic particles (2.7%), metal-containing particles (0.2%), ultrafine particles (UFPs, 30.7%), droplet-like particles (4.2%), and mixed particles (4.5%) (Figure 4). Sulfur-dominant, ultrafine, and mixed particles were the three most abundant types. Detailed descriptions of the physicochemical properties and potential sources of particle type are provided in Table 2.

Soot particles, also called soot aggregates, generally exhibit chain-like (Figure 4c), clustered (Figure 4b), and compact morphologies (Figure 4a). HR-TEM images reveal that these particles comprise numerous carbon spheres (10–150 nm diameter) exhibiting onion-like nanostructures with graphite layers [18]. These combustion-derived particles primarily consist of C and O with minor Si, demonstrate strong hygroscopicity properties, and frequently appear mixed with or absorbed onto other particles. Organic particles (usually called organic matter, OM) display diverse morphologies including spherical, near-spherical, and irregular shapes (Figure 4k), and contain C and O with trace Si, K, and S. OM includes “tarballs”, which are relatively stable under the electron beam and are mostly highly viscous fossil fuel combustion products [12]. Coal-burning experiments have confirmed that the temperature significantly influences the distinct morphological and environmental behavior of these two particle types [19].

Sulfur-dominant particles show diverse morphologies, including spherical, ellipsoidal, elongated, and irregular shapes (Figure 4d–i), with elemental compositions dominated by C, O, and S along with minor Si and Al. Sulfur-dominant particles undergo distinct morphological transformations under electron beam irradiation, with volatile sulfur constituents (primarily sulfates) volatilizing to form foam-like shapes, while more stable components (e.g., carbonaceous and mineral components) remain unchanged (Figure 4f). Sulfur-dominant particles are common in the atmosphere and are primarily derived from the secondary formation of SO_2_ gas, representing key components of secondary inorganic aerosol (SIA) (e.g., SO_4_^2+^, NO_3_^−^, and NH_4_^+^) [20,21]. Therefore, their differential behaviors enable the identification of secondary sulfate particles under electron microscopy. Only a small amount of sulfur-dominant particles originate from primary emissions, such as the combustion of S-containing oil and coal [22]. These SIA particles influence the radiative balance through light scattering and contribute to haze formation via hygroscopic growth mechanisms that increase PM_2.5_ concentrations [23].

Mineral particles mostly possess irregular morphologies (Figure 4j) and consist of C, O, and crustal elements (Si, Al, Fe, Ca, Na, K, and Mg). These particles demonstrate high stability under electron beam irradiation. Some mineral particles may display crystalline structures attributable to the precipitation of water-dissolved salts on collection substrates [24]. Ambient mineral particles primarily originate from dust resuspension (e.g., soil, road dust, and construction activities) and typically exhibit larger particle diameters. Metal-containing particles mainly comprise metallic elements (e.g., Zn, Fe, Cr, and Ca) except C and O, with spherical or irregular morphologies. Their primary sources include industrial emissions, coal-fired power plants, oil refineries, and mechanical wear from vehicle emissions [25]. Ultrafine particles (UFPs), defined as particles with diameters below 100 nm, predominantly form through environmental processes such as fossil fuel combustion, semi-volatile condensation, and industrial emissions [26]. These particles primarily exist in the nucleation mode, with a minor fraction in the accumulation mode, and are generated via collision and condensation mechanisms [27]. Due to their fine particle size, UFPs dominate number concentration but contribute minimally to total mass concentration [28].

Droplet-like particles have low contrast and are hard to locate in TEM images. These particles typically display round droplet shapes, with some containing homogeneously mixed interiors (Figure 4o). Their composition is predominantly C with minor O, Mg, Al, Si, S, and Ca. During EDX analysis, the outer droplet layer demonstrated electron beam stability, suggesting non-volatile or semi-volatile organic matter [29]. Mixed particles consist of at least two distinct particle types (e.g., soot, sulfates, OM, mineral, or droplet-like particles), exhibiting either external mixtures or internal mixture structures that were usually irregular or core–shell structured. Particles with hygroscopic surface properties particularly show enhanced propensity for atmospheric heterogeneous reactions with other particle types.

### 3.3. Variations in Physicochemical Properties of Individual Particles

**Figure 5:**
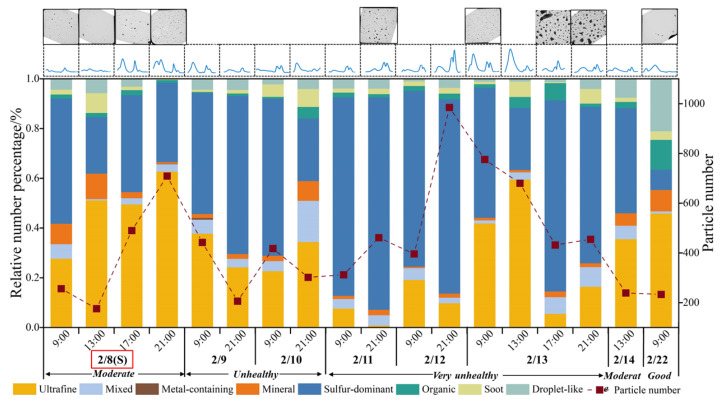
Variations in the relative percentage of particle types, particle number, size distribution, and low-magnification TEM images of deposited particles during the sampling period. Note that sulfur-dominant particles accounted for a high proportion during the polluted days, while they significantly decreased on the cleaner days. The (S) notation of 8 February marked with a red box in the x-axis represents the day of the Lantern Festival. The gray squares and thin blue curves at the top of the graph means the related low-magnification TEM images and size distribution of individual particles.

During the observation period, sulfur-dominant and mixed particles showed relatively higher percentages on “very unhealthy” air quality days and lower percentages on “good” and “moderate” air quality days, while ultrafine particles showed the opposite trends (Figure 5). Organic and soot particles were relatively low, ranging from 2.7% to 2.9% during the lockdown period. Since these particles mainly come from fossil fuel combustion, the low levels suggested that human activities such as traffic and coal-fired emissions were significantly reduced during the lockdown period, which is consistent with other studies that observed low levels of primary organic carbon and elemental carbon during similar emission control events, such as the 2015 China Victory Day [30]. Metal-containing and mineral particles maintained consistently low percentages throughout the observation period, indicating limited contributions from industrial and crustal sources during the observation period. Sulfur-dominant particles varied from 22.7% to 85.5% during the observation period, with a notable increase at 21:00 h on 12 February when the relative humidity significantly increased (Figure 3) and the particle number simultaneously increased (Figure 5). Sulfur-dominant particles were identified by their distinct foam-like morphologies under electron beam and prominent sulfur contents (likely as ammonium and sodium sulfates). These secondary inorganic aerosols (SIAs) were also the dominant components of PM_2.5_, accounting for 50.1%, as proven by Dai et al. [7], which is also consistent with our results. Bulk analyses showed that water-soluble ions (SO_4_^−^, NO_3_^−^, and NH_4_^+^) exhibit marked increases during the heavy pollution days in Beijing [31]. Conversely, the “good” air quality day (22 February) showed significant decreases in sulfur-dominant particles and particle numbers.

**Figure 6:**
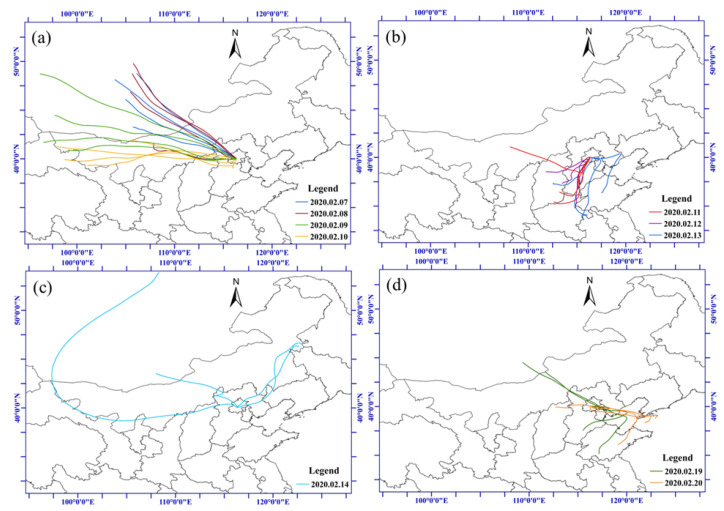
Daily backward trajectories of the sampling sites during the research period. (**a**) backward air masses from 7 to 10 February 2020 (initial pollution phase); (**b**) backward air masses from 11 to 13 February 2020 (intensified pollution phase); (**c**) backward air masses on 14 February 2020 (the pollution removal phase); (**d**) backward air masses from 19 to 20 February 2020 (good air quality days).

Variations in particle size distribution and deposited density are shown in Figure 5, with detailed descriptions in Figures S1 and S2. Air mass transmission during the sampling period was analyzed by the HYSPLIT-4 model (Figure 6) to investigate the long-term transport of air pollutants. Four distinct pollution phases were revealed: (1) Pre-episode phase. The particle size distribution was dominated by the accumulation mode (100 nm < Dp < 1 μm), with particles sparsely distributed. (2) Initial pollution phase. Nucleation mode particles (Dp < 100 nm) gradually increased, peaking on the night of the Lantern Festival firework emissions, indicating increased primary particle emissions with finer particles densely distributed. Air masses mainly came from the northwest and west areas with strong clean winds, indicating that the initial pollution was mainly influenced by the surrounding local emissions. (3) Intensified pollution phase (after 10 February). The percentages of accumulation mode particles increased and became dominant alongside elevated PM_2.5_ concentrations, with coarser particles densely distributed. PM_2.5_ number and mass concentration remained relatively high until the morning of 13 February 2020, when snowfall occurred. Finer particles in the nucleation mode significantly increased as ice nuclei at the initial stage of the snow. Gas precursors (e.g., sulfates, nitrates, and organic compounds) nucleated under high humidity and low temperature, forming new fine particles, resulting in a temporary increase in the number of nucleation mode particles [32,33]. As the relative humidity continued to increase and snowfall intensified at noon on 13 February (Figure 3d), sulfur-dominant particles increased in volume and diameter by hygroscopic growth and increased condensation and collision, leading to an explosive growth of coarser particles and a temporary rise in mass concentrations [34]. In addition, the southern low-speed air mass with more polluted aerosol influenced by the southern urban area also intensified the air pollution. (4) The pollution removal phase (14 February). Subsequently, airborne coarser particles were effectively removed by snowfall via in-cloud and below-cloud scavenging [28], while the air masses rapidly shifted to northward with strong winds, which significantly favored the dispersion of air pollutants. Particle concentration decreased, and the particle size distribution shifted back to accumulation mode, ultimately improving air quality.

## 4. Discussion

### 4.1. The Temporal Evolution and Aging Processes of Individual Particles

The physicochemical characteristics of atmospheric particles, including mixing state, morphology, chemical composition, and particle size distribution, serve as important indicators of heterogeneous and homogeneous reactions occurring during pollution events. Our observation revealed significant temporal variations in particle morphology and size. The most notable transformation was that diverse organic compounds gradually adsorbed onto the “core” particle under conditions of increasing relative humidity and deteriorating air quality. This adsorption process formed distinct coating layers that substantially modified surface properties, ultimately forming core–shell structures. Such morphological transformations are consistent with other large-scale field observation studies demonstrating that “aged” particles typically exhibit either core–shell structures or multi-layered internal structures [35,36]. These structural features provide valuable insights into particle aging processes driven by atmospheric chemical reactions.

**Figure 7:**
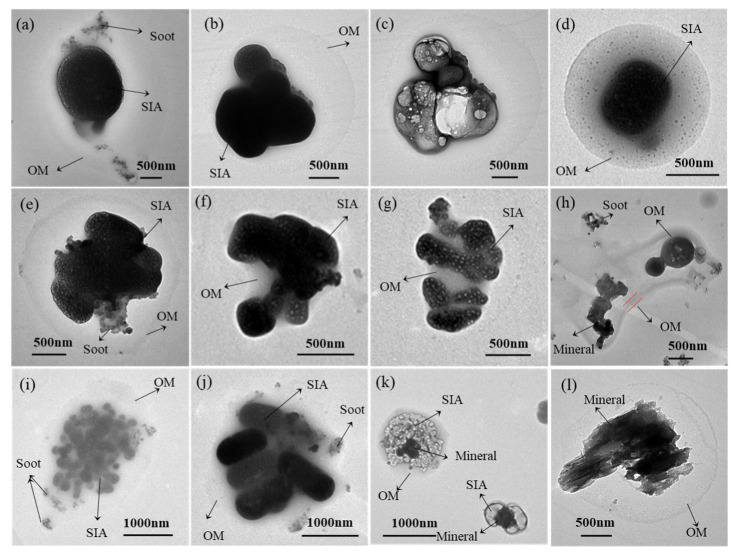
Morphology of core–shell structured particles detected during the air pollution event. (**a**) sulfur-dominant particles (SIA particle for short) externally mixed with soot with a thin OM coating; (**b**) SIA particles with a homogeneously distributed OM outer layer; (**c**) particle; (**b**) after electron beam exposure with the core of SIA particles volatilized and thinner OM outer layer remained; (**d**) regular core of SIA particles with more opaque thick OM outer layer; (**e**) condensed soot internally mixed with SIA particles with a thin outer layer; (**f**) SIA particles with irregular OM coating layer; (**g**) OM internally mixed with SIA particles; (**h**) a larger outer layer of OM combined with soot, spherical OM particles, and mineral particles; (**i**) hollow SIA particles with OM outer layer; (**j**) several rod-like SIA particles and soot with OM coating; (**k**) SIA particles internally mixed with mineral particles and coated with thick OM outer layer; and (**l**) single mineral particle with a thinner OM outer layer.

**Figure 8:**
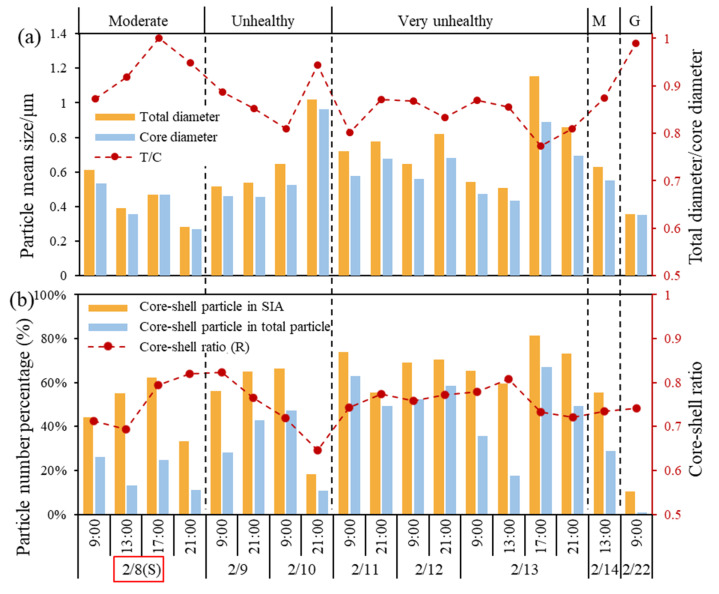
The variations in diameter and core-shell particles. (**a**) Variations in the mean diameter of the particles over the sampling period; (**b**) variation in the proportion of core–shell particles in the total particle and SIA, and the core–shell (R) ratio. Note that the (S) notation of 8 February marked with a red box in the x-axis represents the day of the Lantern Festival.

TEM observations revealed that organic matter, appearing as opaque outer layers, was extensively coated onto various particle types, including sulfur-dominant particles (Figure 7a–g), soot, and mineral particles (Figure 7h–l). The average diameter of the total particle decreased from 0.75 μm on “very unhealthy” air quality days to 0.68 μm on “unhealthy” air quality days, 0.48 μm on “moderate” air quality days, and 0.35 μm on “good” air quality days (Figure 8a). This trend suggests that higher pollution degrees are associated with coarser aged particle sizes. More severe pollution conditions are always coupled with higher particle aging and more frequent secondary growth through atmospheric processing. The particle aging process was further quantified by examining the ratio of core to total diameter, which exhibited the lowest value of 0.82 on “very unhealthy” air quality days, followed by “unhealthy” air quality days (0.89), “moderate” air quality days (0.89–0.91), and “good” air quality days (0.98), and approaching 1 when the shells effectively disappeared. These measurements provide robust evidence that pollution formation is related to shell formation and particle aging processes, with the most significant aging occurring during the heaviest pollution. The reduction in coating thickness and core–shell particles with improving air quality emphasizes the dynamic nature of atmospheric particle formations.

The percentages of core–shell structured particles significantly increased during the pollution process, reaching 10.9–67.1% during the pollution process, while they decreased to below 0.8% on “good” air quality days (22 February) (Figure 8b). This trend suggests that large amounts of gaseous pollutants and particles emitted from fireworks and firecrackers accumulated and continuously underwent physicochemical reactions under adverse weather conditions. This process led to the formation and accumulation of substantial secondary inorganic salts during the pollution process, and core–shell structured particles potentially increased. These processes were further enhanced by high humidity, which promoted the adsorption of organic coating onto the core particles, resulting in increased particle size and elevated PM_2.5_ mass concentrations. Among the sulfur-dominant particles, core–shell particles were always high during the observation period. Sulfur-dominant particles have hydrophilic surface properties that make particles easily combine with organic aerosol. Most of the sulfate particles had an organic coating to a lesser or greater degree on their surface. The proportions of core–shell particles in sulfur-dominant particles were significantly lower on “good” air quality days (22 February). Lower relative humidity and lower primary particle concentrations could reduce the hygroscopicity of sulfate; therefore, the proportion of organic aerosol-coated sulfur-dominant particles would be reduced. Higher relative humidity favors the growth of secondary particles such as sulfate, and the uptake of water by particles under these conditions will increase the mass concentration of particles and directly enlarge particle diameter [20].

To quantitatively evaluate the aging degree of sulfur-dominant particles, we employed the core/shell ratio (R = D core/D particle,), where D core and D particle represent the equivalent circular diameters of the particle core and the entire particle, respectively. A smaller R value (ranging from 0 to 1) corresponds to a thicker sulfate shell and a higher aging degree. As shown in Figure 8b, R exhibited dynamic changes throughout the pollution process (average R = 0.75, range = 0.65–0.82). Notably, R decreased to 0.65 when air quality deteriorated from “unhealthy” to “very unhealthy” (from 10 to 11 February), compared to higher values of 0.78 and 0.76 in the early (8 February) and later stages (14 February), respectively. Despite the lack of significant statistical differences in R, the low proportion of sulfur-dominant particles in the early stage (35.8%) and the later stage (42.3%) suggests weaker sulfate formation and aging process during initial pollution development. Importantly, similar R values were observed at the beginning (8 February: R = 0.76) and end (14 February: R = 0.73) of the pollution episode, as well as on the “good” air quality days (22 February: R = 0.74), indicating suppressed chemical reactions and aging process during these periods.

### 4.2. Phase Separation Variations

**Figure 9:**
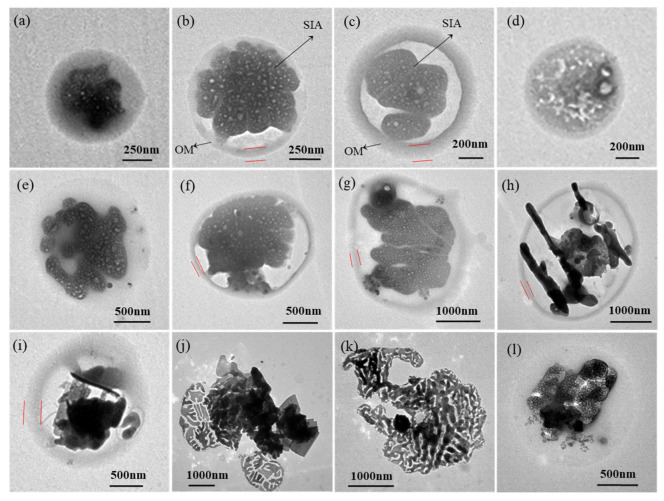
Morphology of “liquid–liquid” phase-separated secondary particles. (**a**) SIA without phase separation with the core and shell coating, showing a distinct shell separation; (**b**,**c**) a joined multiple sulfate particulate core with different degrees of separation; (**d**) a complex sulfate core with “liquid–liquid” separation inside the particle core, showing a distinct shell separation; (**e**) mixed particles with multiple small core components and a distinct outer shell; (**f**–**i**) particles with complex cores consisting of different components such as soot and mineral particles and a separate liquid phase organic shell; and (**j**–**l**) phase separation occurred inside the SIA core with interstitial liquid phases inside voids or joins in the complex cores.

“Liquid–liquid” phase separations between secondary inorganic and organic components were observed during the pollution process, with the inorganic salts as the core and organics as the shell (Figure 9). Three characteristic patterns were observed: (1) homogeneous sulfur-dominant particles without phase separation (Figure 9a); (2) complex sulfate cores (single or aggregated) with clear separation from organic outer coatings (Figure 9b–i); and (3) internally phase-separated sulfate cores with interstitial liquid phases inside voids or joins in the complex cores (Figure 9j–l). Phase separation can easily occur between inorganic sulfur-dominant particles and organic aerosol, either in the form of homogeneously or heterogeneously mixed components. Previous research has demonstrated that phase separation is closely related to the O:C ratio and particle size [37], showing complete separation when the O:C ratio is below 0.56, and serious organic aging when the ratio exceeds 0.8 [38]. Size-dependent trends revealed that 34–55% of particles smaller than 100 nm exhibited “liquid–liquid” phase separation, whereas particles coarser than 100 nm predominantly maintained homogeneously mixed states [23].

Our observations revealed a clear positive correlation between pollution level and the occurrence of phase-separated core–shell particles. The proportions of these particles increased as air quality changed from “good” to “very unhealthy”, with the degree of separation also varying (Figure S3). During the initial pollution period, the separation level increased gradually. Highly phase-separated core–shell particles became dominant during this polluted period. Towards the end of the event, highly phase-separated core–shell particles increased sharply under high relative humidity conditions, with a distinct decline due to the removal effect of snowfall (13 February). These findings highlight the strong dependence of phase separation levels on both air pollutant concentrations and meteorological conditions.

### 4.3. Comparisons Between Highly Polluted and Cleaner Airborne Particles During the Lockdown Period

**Figure 10:**
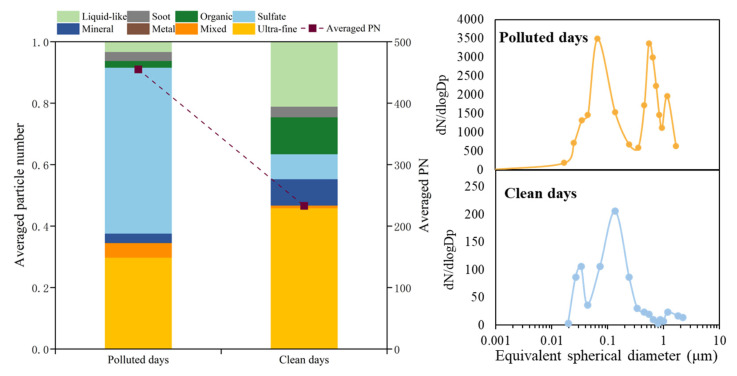
Comparison between polluted days and clean days during the COVID-19 lockdown period. Note that the data are averages, which represent the polluted days and the clean days.

Unbalanced emission reductions coupled with unfavorable meteorological conditions can significantly enhance the secondary formation of air pollutants such as sulfate, even during periods of reduced anthropogenic emissions [39,40]. A comparison between polluted and cleaner days during the lockdown period is shown in Figure 10. Generally, the measured particle number on the cleaner days was significantly lower than that on the polluted days, as well as the distribution density of single particles shown in Figure S2. The percentage of secondary aerosols, such as sulfur-dominant particles (including pure sulfate, sulfate externally mixed with soot, mineral, or other particles), was significantly lower on the cleaner days compared to the polluted days. Secondary inorganic salts became one of the predominant contributors to particle concentration on the polluted days. In terms of primary particles, the proportion of soot particles was also lower on the cleaner days, indicating relatively reduced source emissions, including industrial activities, coal-fired emissions, and vehicle emissions. On the polluted days, the particle size was coarser compared to the cleaner days. There was a notable peak in the coarser-sized fraction on the polluted days, which can be primarily attributed to the aging process of particles. Ultrafine particles show large proportions on the cleaner days, while particles were predominantly larger than 100 nm on the polluted days. When considering the differences in the atmospheric formation processes, the core–shell structured particles significantly decreased on the cleaner days. The above findings evidence that despite overall emission reductions, the degree of the atmospheric aging process can be enhanced with a large proportion of inorganic salts (especially sulfur-dominant particles) explosively formed during the haze episode, especially under unfavorable meteorological conditions.

### 4.4. Atmospheric Implications

Our microscopic observations of individual aerosol particles during the onset of COVID-19 provide direct evidence of the unusual haze episode formation under decreased human activities. They indicate that in the early stage of the haze, the firework emissions increased the proportion of primary fine particles, but the subsequent accumulation and formation of secondary aerosol particles were the primary causes of the further deterioration of air quality with increased PM, especially when the relative humidity increased. The result is consistent with the modeling studies and bulk sample analysis [7,9]. With our observation, the morphological properties of secondary aerosol were directly investigated. It should be noted that the secondary inorganic aerosols were identified as sulfur-dominant particles based on their distinct morphological feature and EDX spectra, and further analysis using advanced techniques is recommended to confirm the definitive evidence of sulfate composition (SO_4_^2−^). This study proves the secondary formation cause of this unusual haze episode and the complicated factors influencing the air quality. It demonstrates that emission reductions alone due to the lockdown measures cannot reduce the occurrence of haze episodes, such as meteorological factors (particularly humidity), atmospheric oxidizing capacity, and regional transportation collectively govern air quality. Our findings underscore the need for integrated pollution control strategies that deal with both primary emissions and secondary formation pathways, maintaining a balanced and coordinated relationship among the influencing factors to improve air quality.

## 5. Conclusions

This study presents a comprehensive microscopic analysis of atmospheric individual particles at the beginning of the COVID-19 pandemic in 2020. Several key findings are given as follows:

(1) Airborne aerosol particles collected during the lockdown period were classified into eight types, including soot (2.9%), sulfur-dominant particles (51.5%), mineral particles (3.3%), organic (OM) particles (2.7%), metal-containing particles (0.2%), ultrafine particles (UFPs, 30.7%), droplet-like particles (4.2%), and mixed particles (4.5%). Sulfur-dominant, ultrafine, and mixed particles were the three most abundant types.
(2) Significantly reduced anthropogenic particles (soot, organic, and metal-containing particles) reflect decreased primary emissions due to inactive source emissions during the lockdown period.
(3) The haze episode during the lockdown period was initially influenced by the firework-derived primary emissions with mostly fine particles, followed by substantial secondary formation, particularly sulfur-dominant particles peaking at 85.5%, and finally removed by precipitation.
(4) Advanced particle aging process was evidenced by increasing core–shell particle proportions (up to 67.1%) and decreasing core/shell ratios (minimum 0.65) during the haze event, demonstrating enhanced secondary inorganic salt formation despite reduced primary emissions.

Our findings demonstrate that the degree of the atmospheric aging process was enhanced with a large proportion of inorganic salts during the haze episode, despite the reductions in anthropogenic emissions at the beginning of the COVID-19 lockdown period. Maintaining improved air quality relies on a balanced relationship among emission reductions, atmospheric oxidizing capacity, and meteorological conditions.

## Figures and Tables

**Table 1:** Information on the collected ambient particle sample.

No.	Date	Air Quality	Duration	PM_2.5_ (μg/m^3^)	PM_10_ (μg/m^3^)	T * (°C)	RH * (%)	P * (hPa)	Note	Weather
1	2/8A *	Moderate (AQI: 82)	2 min 30 s	33.4	43.9	4.1	34.6	1028.8		Sunny
2	2/8N *		3 min	15.7	21.4	12.1	25.5	1026.8		
3	2/8P *		1 min 50 s	73.8	110.5	8.7	30	1026	Firework	
4	2/8E *		1 min	117.8	181.1	6.5	39	1026.6		
5	2/9A	Unhealthy (AQI: 160)	50 s	114.5	155.7	4.1	45	1021.2		Sunny
6	2/9P		20 s	162.3	238.7	15.5	28.9	1017.1		
7	2/10A	Unhealthy (AQI: 163)	35 s	148.4	206.2	12.3	33.4	1016		Sunny
8	2/10P		30 s	116.9	184.2	13	34	1014.6		
9	2/11A	Very unhealthy (AQI: 232)	20 s	218.5	305.9	9.3	42.3	1015.6		Sunny
10	2/11P		12 s	266.5	409.2	11.7	53.9	1013.2		
11	2/12A	Very unhealthy (AQI: 257)	13 s	258.1	368.3	8.3	42.3	1012.3		Haze to fog
12	2/12P		18 s	249.6	346.8	18.5	31.9	1007.8		
13	2/13A	Very unhealthy (AQI: 247)	12 s	262.2	433.7	9.8	40.8	1008.9	Snowfall start	Haze to sleet
14	2/13N		12 s	259.3	381.3	15.3	50.6	1007.2		
15	2/13P		20 s	226.8	346.8	10.2	49.3	1007.8		
16	2/13E		20 s	237.3	379.2	10.9	50.7	1011.4		
17	2/14N	Moderate (AQI: 69)	2 min 40 s	24.9	29.0	11.9	39.7	1020.8	Snowfall finish	Light snowfall
18	2/22A	Good (AQI: 40)	4 min	3.2	4.3	9.3	19.8	1028.7		Sunny

* Note: A represents a.m. (9:00 h), N represents noontime (13:00 h), P represents p.m. (17:00 h), and E represents evening (21:00 h). T represents temperature, RH represents relative humidity, and P represents air pressure.

**Table 2:** Classification and physicochemical properties of individual particle types.

Types	Elemental Composition	Morphology	Stability Under Electron Beam
Soot	Mainly composed of C and O with minor Si and Al	Chain-like or aggregate morphologies with numerous C-rich spheres	Stable
Organic particle	Mainly composed of C and O, with minor Si, K, and S	Spherical, near-spherical, or irregular morphologies	Stable
Sulfur-dominant particle	Primarily composed of C, S, and O	Spherical, near-spherical, or irregular shapes, with foam-like morphologies	Unstable and beam-sensitive
Mineral particle	Mainly composed of Si, Al, Fe, Ca, Na, K, and Mg	Mostly irregular morphologies	Stable
Metal-containing particle	Except C, O, mainly composed of metal elements, including Zn, Fe, Cr, Ca, etc.	Spherical or irregular morphologies	Stable
Ultrafine particle	Mainly composed of C, O with minor Si, etc.	Irregular morphologies	Stable
Droplet-like particle	Mainly composed of C and O, with minor Ca, S, etc.	Mostly round or near-round	Stable
Mixed particle	Complex elements	Irregular morphologies	-

## Data Availability

All the data presented in this paper are available upon request. Please contact the corresponding author (Longyi Shao: shaol@cumtb.edu.cn).

## Supplementary Materials

The following supporting information can be downloaded at: https://www.mdpi.com/article/10.3390/toxics13121051/s1, Figure S1: The variation in the size distribution of the measured particles for each sample during the observation period. Figure S2: Low-magnification TEM images of individual particles during the sampling period. Figure S3: Variation of phase separation among the core-shell particles during the collection interval.
