# Supplementary material for: Microscopic Evidence of Haze Formation During the COVID-19 Lockdown in Beijing: Insights from Physicochemical Properties
Source: Toxics. 2025 Dec 4;13(12):1051. doi: 10.3390/toxics13121051 (PMC12737694; doi:10.3390/toxics13121051)
Supplement: Supplementary file 1 [file toxics-13-01051-s001.zip › toxics-3984363-supplementary.pdf]

Supporting Information for:

Microscopic evidence of haze formation during COVID-19 lockdown in Beijing: Insights from physicochemical properties and meteorological impacts

Wenjun Li[1,2], Longyi Shao[2], Timothy P. Jones[3], Hong Li[1], Daizhou Zhang[4], Weijun Li[5], Jian Gao[1], M. Santosh[6,7], Shushen Yang[8], Kelly BéruBé[9]*

[1] State Key Laboratory of Environmental Criteria and Risk Assessment, Chinese Research Academy of Environmental Sciences, Beijing 100012, China

[2] College of Geoscience and Surveying Engineering, China University of Mining and Technology (Beijing), Beijing 100083, China

[3] School of Earth and Environmental Sciences, Cardiff University, Cardiff, CF10, 3YE, Wales, UK

[4] Faculty of Environmental and Symbiotic Sciences, Prefectural University of Kumamoto, Kumamoto 862-8502, Japan

[5] Department of Atmospheric Sciences, School of Earth Sciences, Zhejiang University, Hangzhou 310027, China

[6] School of Earth Sciences and Resources, China University of Geoscience Beijing, Beijing 100083, China

[7] Department of Earth Science, University of Adelaide, Adelaide SA 5005, Australia

[8] Department of Environment Engineering, Zhongyuan University of Technology, Zhengzhou 450007, China

[9] School of Biosciences, Cardiff University, Cardiff, CF10 3AX, Wales, UK

Correspondence to: Longyi Shao (ShaoL@cumtb.edu.cn)

Figures: Figure S1 to Figure S3

Figure S1. The variation in the size distribution of the measured particles for each sample during the observation period.

Figure S2. Low-magnification TEM images of individual particles during the sampling period.

Figure S3. Variation of phase separation among the core-shell particles during the collection interval.

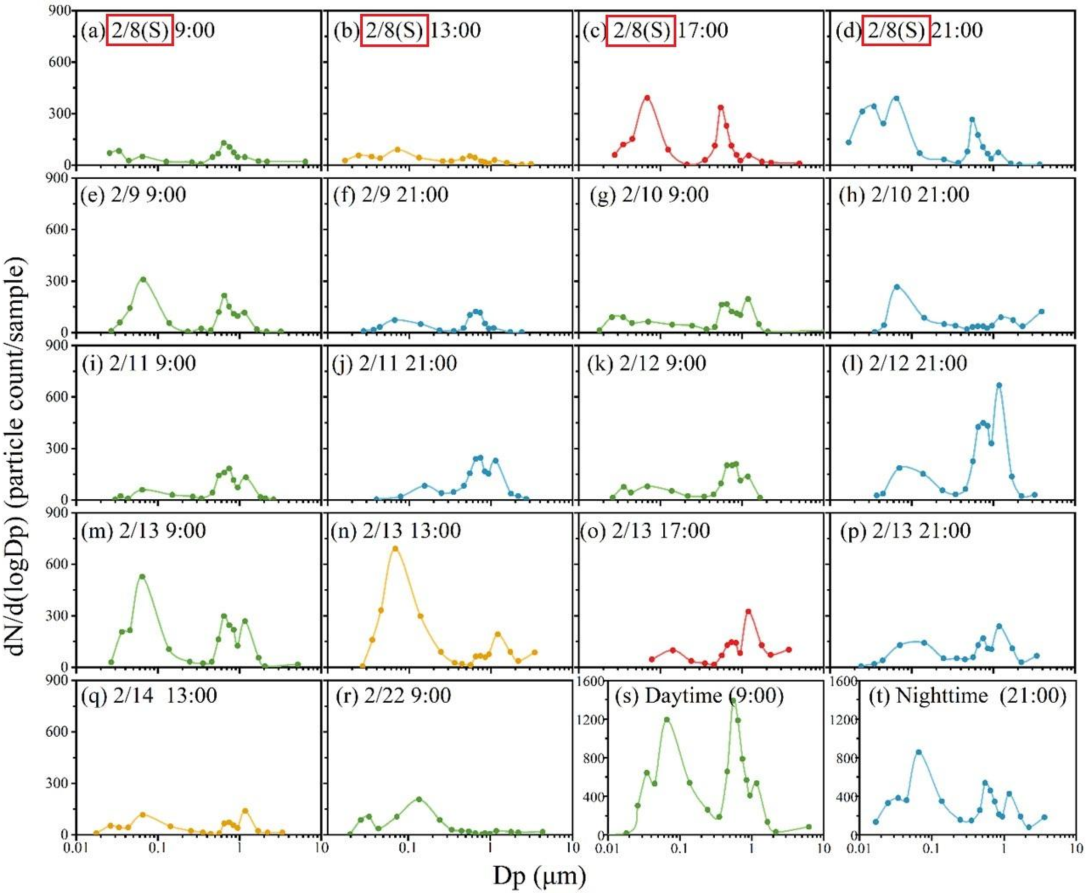

Figure S1. The variation in the size distribution of the measured particles for each sample during the observation period. Note that the primary emission increased the peak of the nucleation mode value. With the pollutants accumulated, the particle size increased with a decrease in nucleation mode and an increase in accumulation mode, and then the particle concentration decreased by the precipitation. The (S) notation of February 8th marked with red box after the date represents the day of the Lantern Festival.

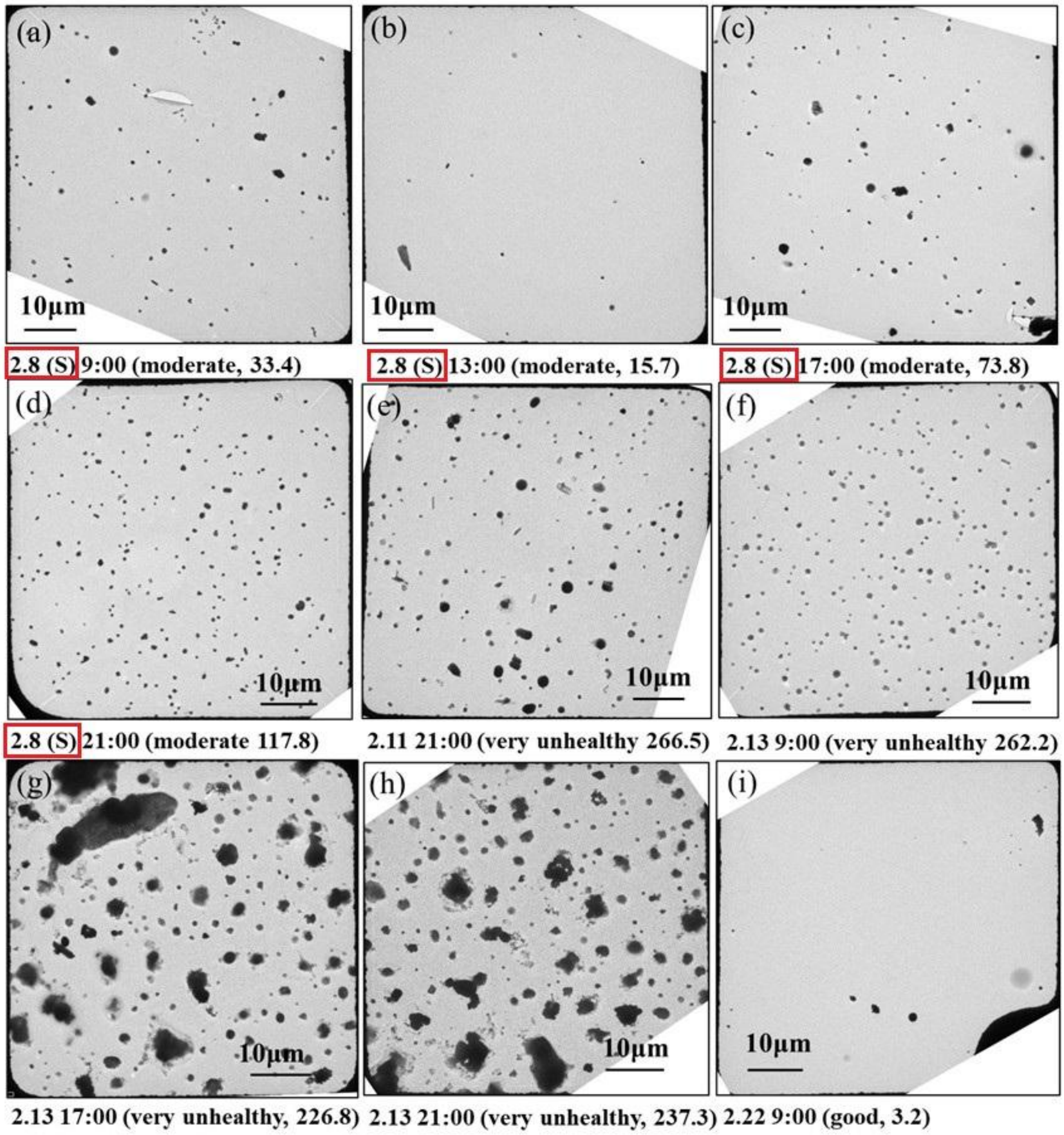

Figure S2. Low-magnification TEM images of individual particles during the sampling period. Note that the footnote at the bottom of each picture is the information about the sample period and air quality, such as (a) means the sample was collected at 9:00 hrs on February 8th when the air quality was moderate and the $PM_{2.5}$ concentration was 20 μg/m^3. The samples were dominated by fine primary particles at the beginning of the sampling period, which gradually accumulated over time. Sulfate particles had significant growth with larger sizes, and the period ended with their wet deposition. The (S) notation after the date of February 8th marked with red box represents the day of the Lantern Festival.

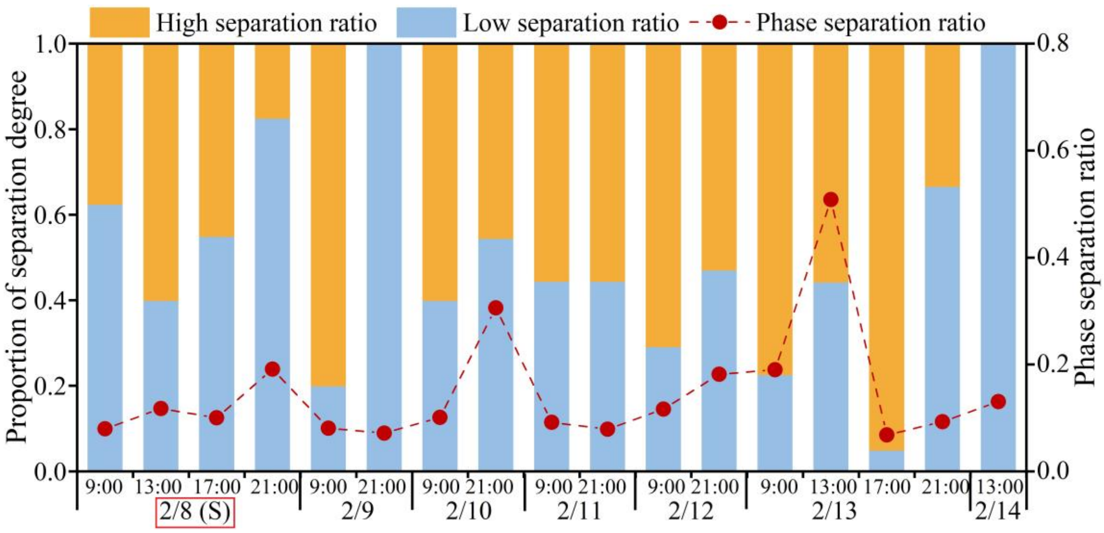

Figure S3. Variation of phase separation among the core-shell particles during the collection interval. Note that the core-shell particles with phase separation increased with the increase of pollution levels, and the separation degree of particles was related to the air pollution levels. The (S) notation of February 8th marked with red box in the x-axis represents the day of the Lantern Festival.
